# The Practice Market

**Published:** 1911-03-18

**Authors:** 


### SPECIAL ARTICLES.

### THE PRACTICE MARKET.

### I.—INTRODUCTORY ARTICLE.

Some men are born general practitioners, with family practices waiting for them to drop into as soon as they are qualified; others achieve success in general practice after patient years of plodding behind a brass plate; while others again have general practice thrust upon them by force of circumstance and against their original intentions. But the majority enter the arena of private practice through some business gateway, presided over by a medical agent. And in whatever manner he may have entered upon practice, it is almost certain that every general practitioner will come into direct contact, at least once in his career, with what we may call the Practice Market. For, when all is said, every general practice is essentially a business undertaking, and so long as a single patient remains upon its books it continues to be a saleable commodity.

Scope of the Articles.

In this and several future articles we intend to discuss, in considerable detail and from more than one point of view, the business side of the transfer of medical practices and partnerships. We have pointed out that this is a matter which at some time or another touches very closely the interests of almost every general practitioner. The Practice Market is full of difficulties and dangers, not only to those who buy, but also to those who sell. It is obvious that the selection of a sound opening is a matter of vital importance to the junior man about to invest his capital in general practice. And to the practitioner already established, whose work is getting beyond his unaided powers, the choice of a satisfactory partner is scarcely less fraught with anxiety and responsibility. Even to the elderly doctor upon the verge of retirement the discovery of a *bonâ-fide* purchaser, who will behave honourably towards himself and his old patients, is not altogether simple and easy.

Medical Agents.

We have thought the matter well over and have made many inquiries, and it seems to us that the whole subject of medical transfer needs thorough ventilation. The medical profession as a whole is notoriously bad at business. For lack of proper guidance past the commoner pitfalls which beset them in the Practice Market, hundreds of otherwise capable medical men each year come to grief. We have no special quarrel with the medical agents. They are business men and they have their living to make, and their methods, so far as we can discover, differ little from those of other men of business. For their own reputations' sake the better class of medical agents try to do their best for their clients. But their main object, quite naturally, is to effect a sale, and their motto is *Caveat emptor*.

The Business Side of Practice.

The average medical student knows nothing at all about the business side of practice, and less than nothing about the mechanism of the Practice Market. He has to buy his own experience in both directions after qualifying, and he often buys it very dearly. It is a pity that he receives no instruction at all in these important matters whilst he is at hospital, but in the present loaded state of the curriculum it is difficult to suggest a remedy.

But it is not only among the newly-qualified that ignorance prevails about the technique of medical transfer and about the traps and stumbling-blocks which assail the unwary at every turn in the Practice Market. Many competent practitioners of ripe experience and sound business judgment, when it comes to replacing a partner or disposing of a share or the whole of their practice, display the most amazing ignorance of how to go about the matter, and are quite as likely to do injustice to themselves as to the other party to the negotiations.

The Market.

The fact is that the sources of information upon the methods and dangers of the Practice Market are limited, not readily available, and little known to the ordinary medical man. Those who have been through the protracted misery of a "practice hunt" know that practices and partnerships are handled and transferred by agents upon well-established lines, and that negotiations for sales and divisions are carried out upon a definite basis. We have inquired closely into these and allied matters, and we propose to discuss every aspect of the Practice Market, both from the vendor's and the purchaser's standpoints.

